# 

**Published:** 1912-06-15

**Authors:** 


### ANNOUNCEMENTS.

Northern Ohio Dental Association.—The annual meeting of this society will be held June 11—13 at Cedar Point, O., a fine resort near Sandusky, O. From the programme this promises to be one of the most interesting and profitable meetings held anywhere this spring. It will have a most delightful place of meeting if the weather is propitious.

West Virginia State Society.—The annual meeting of this society will be held at Webster Springs, August 14th to 16th. The time and place ought to attract a large attendance. Dentists from all adjoining States and in fact all ethical practitioners from anywhere are invited and will be cordially welcomed.

Dr. F. L. Wright, *Secy.*, Wheeling, W. Va.

North Dakota Examiners.—The next regular session of this Board will be held July 9th to 12th at Fargo. For particulars address

Dr. F. A. Bricker, Fargo.

Idaho Examining Board.—This Board will hold its next examination in Boise City, beginning July 1st, 1912.

Albert A. Jessup, *Secy.*, Boise City, Idaho.

American Society of Orthodontists.—The annual meeting of this society will be held in Chicago, July 1st to and including the 3rd. This promises to be one of the best meetings of this society and a cordial invitation is extended to all practitioners.

Dr. F. C. Kemple, 576 5th Ave., N. Y., *Secy.*

North Carolina State Board of Examiners will hold its next session for registration at Raleigh, July 1st, 1912.

Dr. F. L. Hunt, Asheville, N. C., *Secy.*

---

Vermont Examining Board will hold its annual session at Montpelier, July 1—3, 1912. For particulars address

Dr. G. F. Teney, *Secy.*, St. Johnsbury, Vt.

---

New Jersey State Dental Society. The annual meeting of this society of which Dr. Meeker is Secretary, will hold its session at Cape May, N. J., July 17—19, in the Hotel Cape May. The papers to be read this year are very interesting.

Thomas Bliss Stillman, M. Sc. Ph. D., of Stevens Institute, "Ancient and Modern Alchemy."

Dr. W. W. Belcher, Editor of "Dispensary Record," Rochester.

Dr. Herbert L. Wheeler, President of the First District of New York Society.

H. L. Ambler, M. Sc. D. D. S., Cleveland, Ohio, A Travellogue Around of the World of Dentistry—More than interesting.

Dr. George H. Wilson, of Cleveland, Prosthetic Dentistry.

Dr. George Evans, of New York.

S. B. Harris, D. D. S. and A. J. Quinby, D. D. S., of New York, Radiography.

Dr. Thomas Dedrick, of Peary Polar Exploration. Meningitis.

Dr. W. J. Roe, of Philadelphia, Tic Doloroux.

A WALIDICTORY by Charles A. Meeker, D. D. S. A five minute paper on thirty-seven consecutive years of official service in the New Jersey State Dental Society.

EXHIBITS. More space than ever before. More varied and up-to-date. Some space still left. Write Dr. Moore Stevens 1503 Pacific Avenue, Atlantic City, N. J., for floor plans.

CLINICS. The Latest in Dentistry, with Dr. M. R. BRINKMAN, of Hackensack, N. J., in charge.

Automobile and power boat races, hot salt water baths in hotel, garage, golf links. Come and spend a week. Write the hotel for rooms.

Reached by Pennsylvania and Central Railroads.

Charles A. Meeker, D. D. S., Secy., 29 Fulton St., Newark, N. J.

We do not know where the other section will hold its session. If you are interested write to Dr. T. J. McLernon, Camden, N. J., for information.

---

ATTENTION.—MEMBERS OF THE DENTAL PROFESSION OF THE UNITED STATES OF AMERICA.—The XV. International Congress on Hygiene and Demography is to be held in Washington, D. C., September 23—28, 1912, under the auspices of the United States Government.

This is the most important meeting of this kind held in this country in its history; and the United States Government is acting as host to the fifteen nations that have so far signified their intention of participating in the coming Congress.

This organization is the highest authority in matters of Hygiene in existence to-day.

Through the courtesy of the United States Government the Dental Profession of this country has received an invitation to contribute to the success of the coming Congress. A place has been made for representatives of the Dental Profession both upon the literary program and among the exhibitors. This is the first time that the dental profession of this country has received such recognition by the home Government.

The opportunity for which we have been seeking, that is, the opportunity to show the important relation the human mouth bears to the health, strength and welfare of mankind is now before us.

The influence of this Congress is world wide in its scope and will be visited by thousands upon thousands of people who are interested in Hygiene and the general welfare of mankind.

If American Dentistry is to maintain its reputation throughout the world it behooves the members of the profession of this country to unite in a general effort to have the largest, finest and most instructive dental exhibit in the history of dentistry assembled on this occasion.

At the request of the Oral Hygiene Committee of the National Dental Association, Dr. J. W. Schereschewsky, U. S. P. H. and M. H. S., Director of the Exhibition, has set aside 1000 square feet of floor and 500 square feet of wall space in the building, which is being erected for the exhibits, to be devoted for the use of the dental profession for exhibit purposes.

At the meeting of the Oral Hygiene Committee of the National Dental Association, held in Cleveland, March 23rd, 1912, a resolution was passed inviting the Oral Hygiene Committees of all State and Local organizations to cooperate with it in making a success of this exhibit. Space will be assigned in such a manner that each State, City and Town will receive full credit for contributions in this direction.

The Committee earnestly requests that every member of the profession, who is interested in Mouth Hygiene and the welfare of the dental profession, become actively interested in a campaign to make a success of this exhibit. The Oral Hygiene Committee of the State Dental Societies should endeavor to place themselves in touch with local organizations in their States in an endeavor to secure aid in the way of material suitable for exhibits and in money to defray the expenses of such an exhibit as this should be. The Committee would ask that each State and Local organization make appropriations to meet the expense of collecting, mounting and displaying such material as would make a creditable exhibit.

The Committee requests that the Oral Hygiene Committees that can or will take part in this exhibit, communicate at once, or at the earliest possible moment with Dr. W. G. Ebersole, Chairman of the Oral Hygiene Committee of the National Dental Association, 800 Schofied Building, Cleveland, Ohio, or, for local information to Dr. W. Smith Frankland, The Burlington, Washington, D. C., Assistant Sec'y-Treas. of the National Mouth Hygiene Association for the District of Columbia.

The Oral Hygiene Committee of the National Dental Association instructed its Secretary, Dr. Waldo E. Boardman of Boston, Mass., to communicate with Dr. William H. Porter of Boston, Mass., with a view of obtaining some idea of the dental exhibit which was shown at the International Hygiene Exhibition in Dresden, May—October, 1911. Dr. Potter's letter is given herewith with a view of giving some idea of how to build or prepare an exhibit of this kind.

"Boston, April 13th, 1912.

Dear Doctor Boardman:

In regard to the Dental Exhibit at the International Hygiene Exhibition in Dresden May—October 1911, I am obliged to rely on my memory in as much as I was unable to find a catalogue of this portion of the department. There were in the exhibit as follows:

1. Large numbers of anatomical specimens. Skulls: parts of skulls with teeth in place. In this respect it was similar to the exhibition in connection with the fifth International Dental Congress at Berlin 1909.

2. Orthodontia cases represented by models. Regulating apparatus.

3. Teeth representing the progress of decay from the initial softening to the large destructive cavity.

4. Charts showing the percentage of dental decay amongst people of various occupations and living under various conditions.

5. Charts showing the influence of food and water (hard or soft) upon the percentage of dental decay.

6. Charts giving rules for the prevention of decay.

7. School dental clinics. A description of the most important ones of Europe with literature giving statistics and methods of work.

8. The analysis of saliva. Charts showing the method employed.

These are a few of the features. There were many more which I wish I could remember.

Very truly yours,

WILLIAM H. POTTER."

Let every member of the profession who is interested write, offering to do his part. Do not wait for us to write to you, for we have much to do if we undertake to make a success of this work.

At the same meeting, the Oral Hygiene Committee of the National Dental Association passed a resolution inviting the Dental Colleges of this country to contribute to the success of the dental exhibit; and the Secretaries or Deans of the various colleges are requested to communicate either with Dr. W. G. Ebersole or Dr. W. Smith Frankland indicating what aid they will give in connection with the coming exhibit. The exhibit will be so arranged that each college will be assigned space for its own exhibit.

Come to our aid and give us your hearty support in this work.

Appealing to every member of the profession to become actively interested in this exhibit in the interest of the dental profession as a whole, we are,

Respectfully yours,

THE ORAL HYGIENE COMMITTEE OF
THE NATIONAL DENTAL ASSO.
W. G. EBERSOLE,
B. HOLLY SMITH,
WALDO E. BOARDMAN,
J. W. CONZETT,
S. W. FOSTER.

